# A Novel Universal Primer-Multiplex-PCR Method with Sequencing Gel Electrophoresis Analysis

**DOI:** 10.1371/journal.pone.0022900

**Published:** 2012-01-17

**Authors:** Wentao Xu, Zhifang Zhai, Kunlun Huang, Nan Zhang, Yanfang Yuan, Ying Shang, Yunbo Luo

**Affiliations:** 1 Laboratory of Food Safety, College of Food Science and Nutritional Engineering, China Agricultural University, Beijing, China; 2 The Supervision, Inspection and Testing Center of Genetically Modified Food Safety, Ministry of Agriculture, Beijing, China; University of Delhi, India

## Abstract

In this study, a novel universal primer-multiplex-PCR (UP-M-PCR) method adding a universal primer (UP) in the multiplex PCR reaction system was described. A universal adapter was designed in the 5′-end of each specific primer pairs which matched with the specific DNA sequences for each template and also used as the universal primer (UP). PCR products were analyzed on sequencing gel electrophoresis (SGE) which had the advantage of exhibiting extraordinary resolution. This method overcame the disadvantages rooted deeply in conventional multiplex PCR such as complex manipulation, lower sensitivity, self-inhibition and amplification disparity resulting from different primers, and it got a high specificity and had a low detection limit of 0.1 ng for single kind of crops when screening the presence of genetically modified (GM) crops in mixture samples. The novel developed multiplex PCR assay with sequencing gel electrophoresis analysis will be useful in many fields, such as verifying the GM status of a sample irrespective of the crop and GM trait and so on.

## Introduction

Nucleic acid analysis has become increasingly important in a variety of applications, such as the genotyping of individuals, the detection of infectious diseases, tissue typing for histocompatability, identifying individuals in forensic diagnosis, paternity testing, and monitoring the genetic make-up of plants and animals in agricultural breeding programs [1]. Techniques based on polymerase chain reaction (PCR) provide a powerful tool for the amplification of minute amounts of initial target sequences. Most PCR protocols involve reactions that amplify a single target. Multiplex PCR is a variation of the conventional technique in which two or more targets are simultaneously amplified in the same reaction. This approach has the potential for greater reliability, flexibility, and cost reduction. As far as we know, nine-target multiplex PCR method has been reported to simultaneously detect eight maize lines as well as the endogenous *Zein* gene in a single reaction tube [2], which contains the most targets in reported multiplex-PCR methods.

Multiplex PCR is an essential cost-saving technique for large scale scientific, clinical, and commercial applications, such as infectious microorganisms detection [3], gene expression [4], [5], whole-genome sequencing [6], forensic analysis including human identification and paternity testing [7], the diagnosis of infectious diseases [8], and pharmacogenomic studies aimed at understanding the connection between individual genetic traits, drug response and disease susceptibility [9], [10]. In recent years, multiplex PCR has emerged as a core enabling technology for high-throughput SNP genotyping [7], [10].

With the rapid development of GM crops, more and more studies have recently described the use of multiplex PCR as a rapid and convenient screening assay for the detection of GMOs. In GM crops such as soybean, maize, and canola, a multiplex PCR system has been developed to detect multiple target sequences using simultaneous amplification profiling [11]. A sensitive and specific triplex nested PCR assay was developed for the detection of housekeeping gene (lectin) and inserted elements of Roundup Ready soybean, i.e., constitutively expressed *CaMV 35S* promoter, *Cp4 epsps* gene encoding for 5-enol-pyruvyl-shikimate-3-phosphate for herbicide tolerance, *nos* terminator, and a chloroplast transit peptide (ctp) facilitating transport of epsps protein, in highly processed products [12]. Multiplex PCR simultaneously detecting eight lines of GM maize by employing sequence-specific primers and the maize endogenous *Zein* gene was developed [2], which is also a most targets multiplex PCR system nowadays. Recently, multiplex PCR assays simultaneously amplifying the commonly used selectable marker genes, i.e., *aadA*, *bar*, *hpt*, *nptII*, *pat*, and a reporter gene *uidA* were developed as a reliable tool for qualitative screening of GM crops [13]. What's more, multiplex PCR-based assays have also been developed to simultaneously detect functional transgenes, control elements and housekeeping genes, such as *cry1Ac* gene for insect resistance, *CaMV 35S* promoter and endogenous *SRK* (S-locus Receptor Kinase) gene in Bt cauliflower [14]; *osmotin* gene for salinity and drought tolerance, *CaMV35S* promoter and endogenous *LAT52* (late anther tomato) gene in GM tomato[15]; *cry1Ab* gene for insect resistance, *CaMV 35S* promoter; *npt II* marker gene and endogenous *UGPase* (uridine diphosphate glucose pyrophosphorylase) gene in Bt potato[16]. These studies demonstrate that the multiplex PCR system is also a convenient, cost-effective, and efficient assay for GM detection.

Although multiplex PCR has so many advantages, it has several disadvantages that can not be ignored, mainly including the self-inhibition among different sets of primers, low amplification efficiency and no identical efficiency on different templates, which restricts its further development and broad application. Even the reported nine-target multiplex PCR method cannot avoid the disadvantage of worse reproducibility and stability. A novel universal primer-multiplex PCR (UP-M-PCR) method was devised at the basis of the problem originating from conventional multiplex PCR, and up to now, it has been used to simultaneously detect four meat species including chicken, cattle, pig and horse [17], to simultaneously detect *Escherichia coli*, *Listeria monocytogenes*, and *Salmonella* spp. in food samples [18], and used in the event-specific detection of stacked genetically modified maize Bt11×GA21 [19]. In the present study, we also use this novel universal primer-multiplex PCR method to simultaneously detect nine commonly used selectable marker and reporter genes, including *hpt*, *gus*, *nptII*, *aadA*, *pat*, *35s*, *bar*, *nos*, *uidA*, as well as six endogenous genes *Pa*, *Ivr*, *Lec*, *sps*, *sad1* and *FatA* of six most common GM crops papaya, maize, soybean, rice, cotton and canola, respectively, which could overcome the shortcomings of conventional multiplex PCR described previously, and significantly simplify the procedure of identification for GMOs. For the analysis of PCR products, sequencing gel electrophoresis (SGE) was chosen as its large separation scale and extraordinary resolution, which could not restrict the increase of detecting targets in multiplex PCR. The fifteen-target UP-M-PCR we developed not only contains the most targets in all the multiplex PCR methods reported before, but also covers most selectable marker and reporter genes commonly employed in GM crops, which will totally meet the demand of verifying the GM status of a sample irrespective of the crop and GM trait.

## Results

### Specificity of Compound Specific Primers

**Figure 1:**
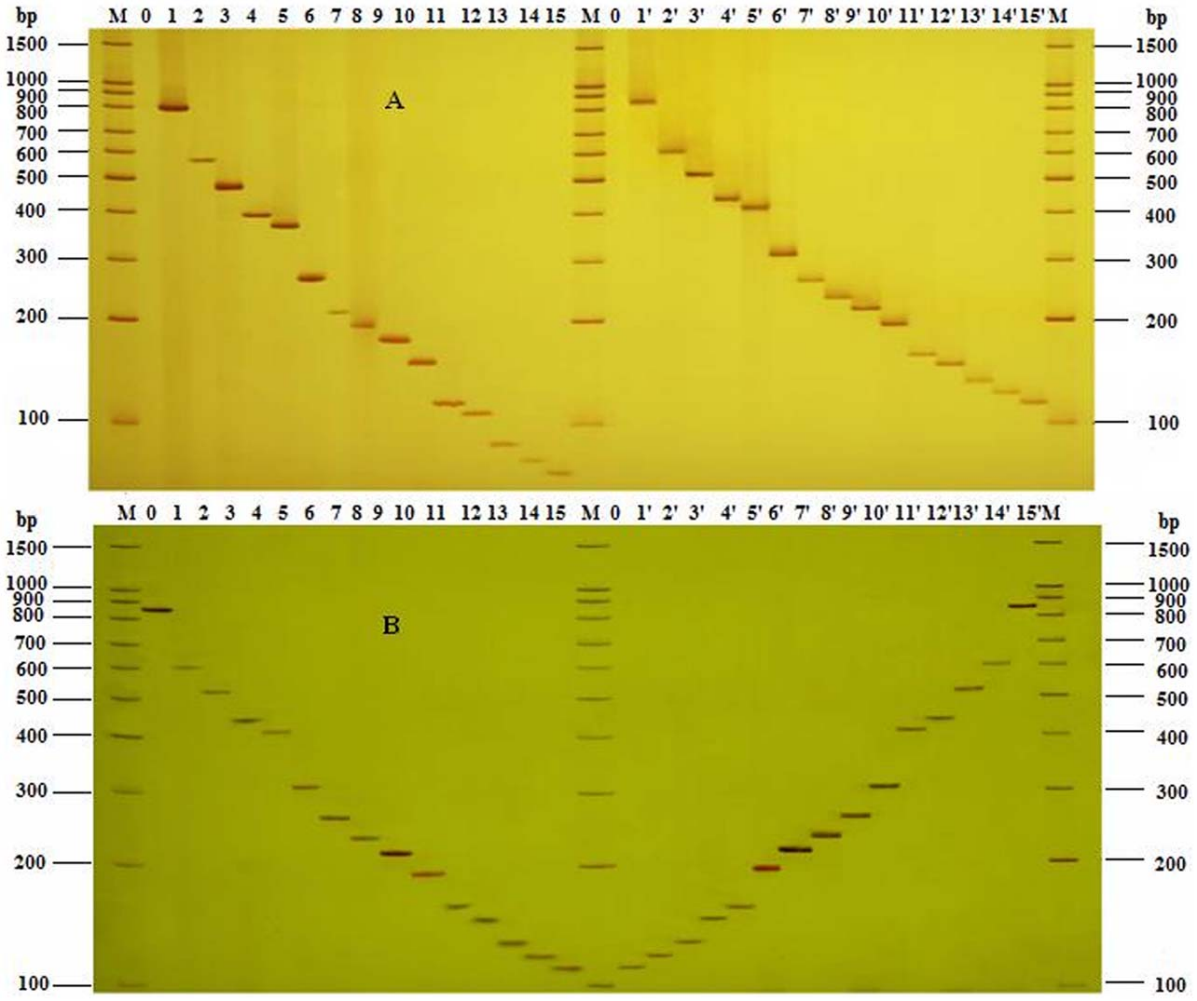
Detection of the specificity of UP-M-PCR. (**A**) Comparison of specificity between specific primers and compound specific primers. Lanes 1∼15, amplicon fragments of specific primer; lanes 1′∼15′, amplicon fragments of UP and compound specific primer; lane 0/0′, negative control without template; lane M, 100 bp DNA Marker. (**B**) Lanes 1∼15, amplicon fragments of one specific primer in singlet PCR; lanes 1′∼15′, amplicon fragments of fifteen-plex PCR with only one certain template DNA; lane M, 100 bp DNA Marker.

The new designed compound primer pairs originated from specific primers have been tested to get equivalent intensities of bands on gels with the same template concentration (Figure 1A), which showed that the set of compound specific primers worked efficiently and had the same specificity as the specific primers from reference. Because the compound specific primers contained a common sequence (20 bp) at the 5′-end, as a result they got a higher annealing temperature and generated amplicons larger of 40 bp than the products amplified by corresponding specific primers, so the bands on gel were a little higher too. In fifteen-plex PCR, all the primers mixed together with the optimized concentration, while the template of each primer pair was added separately in every single reaction, and only one expected PCR amplicon was achieved corresponding to a certain template DNA (Figure 1B). That there were no unexpected bands showed there was no unexpected reaction, which also proved that the specificity of compound specific primers was high.

### Feasibility of Universal Primer (UP)

**Figure 2:**
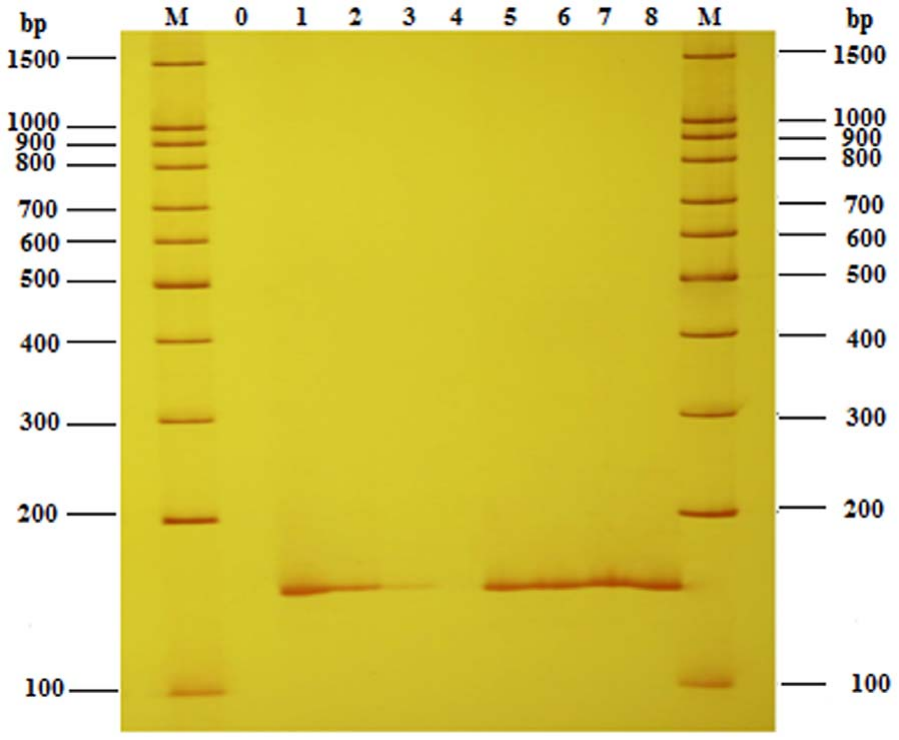
Impact of concentration of universal primer on singlet PCR. Lane 0, negative control without template; lanes 1, 2, 3, 4, amplicon fragments by compound specific primer pair Lec-118-F/R at the concentration of 500 nmol L^−1^, 50 nmol L^−1^, 25 nmol L^−1^, 5 nmol L^−1^ respectively; lanes 5, 6, 7, 8, amplicon fragments by UP (500 nmol L^−1^) and compound specific primer Lec-118-F/R at a series concentrations of 500 nmol L^−1^, 50 nmol L^−1^, 25 nmol L^−1^,5 nmol L^−1^; lane M, 100 bp DNA Marker.

Keeping the concentration of templates at 50 ng, with the amount of the specific primers for *Lec* gene decreasing (500 nmol L^−1^, 50 nmol L^−1^, 25 nmol L^−1^, 5 nmol L^−1^ ), the intensity of band fell down markedly (25 nmol L^−1^) until to nothing (5 nmol L^−1^) in conventional singlet PCR (Figure 2, lanes 1, 2, 3, 4), which showed that the concentration of amplified fragments became lower and lower. While in the novel singlet PCR, for the addition of universal primer (500 nmol L^−1^), though there is a down gradient concentration of compound specfic primers Lec-118-F/R from 500 nmol L^−1^ to 5 nmol L^−1^, the PCR system above worked efficiently and got an equivalent amount of amplified products (Figure 2, lanes 5, 6, 7, 8). Similar results were achieved from other compound specific primers (data not shown) with UP in novel singlet PCR. The sharp contrast showed that the universal primer was well designed to work efficiently for the PCR amplification and had a high feasibility to amplify the amplicons produced by compound specific primers.

### Optimization of the UP-M-PCR

**Figure 3:**
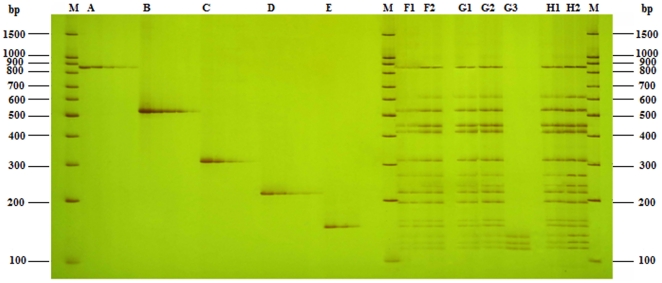
Optimization of the UP-M-PCR. Lane A, B, C, D, E, amplicon fragments by UP (500 nmol L^−1^) and compound specific primer hpt-839, nptII-508, pat-262, bar-226 and sps-110 at a series concentrations of 500 nmol L^−1^, 50 nmol L^−1^, 25 nmol L^−1^, 5 nmol L^−1^, 0.5 nmol L^−1^; lane F1, amplicon fragments by UP at 500 nmol L^−1^ and all compound specific primers at 25 nmol L^−1^; lane F2, amplicon fragments by UP at 500 nmol L^−1^ and all compound specific primers at the optimized concentration; lane G1,G2,G3, amplicon fragments by all primers at the optimized concentration with *TaKaRa Taq*™, Phire™ Hot Start DNA polymerase, iProof™ High-Fidelity DNA polymerase; lane H1, amplicon fragments by all primers at the optimized concentration with Phire™ Hot Start DNA polymerase under the common amplification conditions; lane H2, amplicon fragments by all primers at the optimized concentration with Phire™ Hot Start DNA polymerase under the optimized amplification conditions; lane M, 100 bp DNA Marker.

**Figure 4:**
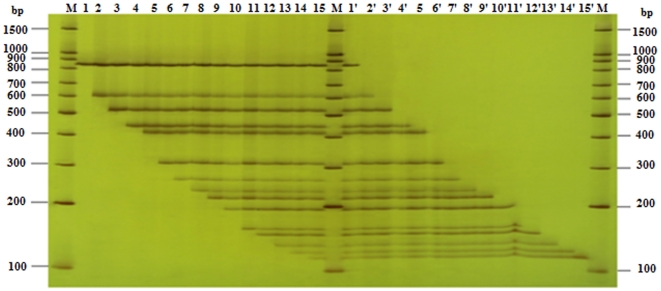
Multiplex PCR assay for testing of primer interference. Using equivalent DNA mix of six different GM events including Bt11 of maize, MON 15985 of cotton, GM rice with *bar* gene, Huanong No. 1 of papaya, RRS of soybean and GM canola with *hpt* gene. Lanes 1∼15, PCR runs starting with the largest amplicon (by *hpt* gene-specific primer, amplicon size 879 bp), followed by the addition of a second primer pair, until the fifteenth primer pair; lanes1′∼15′, PCR runs starting with the fifteen-plex amplicon, followed by the elimination of the largest amplicon primer pair (by *hpt* gene-specific primer, amplicon size 879 bp), until only the smallest amplicon (by *FatA* gene-specific primer, amplicon size 116 bp) remained; lane M, 100 bp DNA Marker.

The concentrations of primers strongly influence the efficiency and disparity of PCR reaction, which is very important for the PCR reaction, especially in multiplex PCR. The final optimized concentration of universal primer (UP) was 500 nmol L^−1^ in both singlet PCR and UP-M-PCR, which is the same as in normal singlet PCR, while the compound specific primers were 25 nmol L^−1^ (about 1/20 of UP) in singlet PCR that can ensure an efficient amplification for all these primers, but in fifteen-plex UP-M-PCR, because of the interaction and the difference of work efficiency among primers, all compound specific primers at 25 nmol L^−1^ could not get an equivalent amount of amplified products, thus there need an adjustment. The process of the adjustment was showed in Figure S2. The final optimized concentration of the compound specific primers were 10 nmol L^−1^ for 35s-195-F/R and Nos-F/R (about 1/50 of UP), 16 nmol L^−1^ for nptII-508-F/R and Pa-363-F/R(about 1/30 of UP), 50 nmol L^−1^ for hpt-839-F/R, Ivr-262-F/R and Lec-110-F/R(about 1/10 of UP), and 25 nmol L^−1^ for all other primers (including gus-565-F/R, aadA-406-F/R, pat-262-F/R, bar-177-F/R, sps-110-F/R, sad1-91-F/R, uidA-82-F/R and FatA-76-F/R). To test the efficiency of Taq Polymerase to be employed in PCR assays, comparative tests were made with several Taq polymerases, such as Phire™ Hot Start DNA polymerase, iProof™ High-Fidelity DNA polymerase, and *TaKaRa Taq*™. The Phire™ HotStart DNA polymerase, coupled with a preoptimized primer mix for different multiplex reactions, gave the best results both in terms of reproducibility and robustness. To find the best annealing temperature, a gradient temperature PCR from 56 to 64°C has been performed. At last the optimum annealing temperature was chosen at 60°C. Similarly the best extension temperature was chosen at 70°C The time for annealing and extension was chosen at 50 s. Figure 3 showed the amplification results by UP-M-PCR of the optimizing process on 5.0% polyacrylamide gel (3.5 M urea included). All the reactions were performed with the same amount of template (50 ng for each). Each compound specific primer pair in the mixture was sensitive and specific enough to amplify the corresponding sequence and generated the expected length of amplicons the same as in the singlet PCR and no unexpected PCR products were detected. There was less or even no disparity between various primers as in UP-M-PCR. Similar duplex, triplex, four-plex, even thirteen-plex or fourteen-plex PCR results were achieved with arbitrary combination of compound specific primer pairs (Figure 4).

### The sensitivity of UP-M-PCR

**Figure 5:**
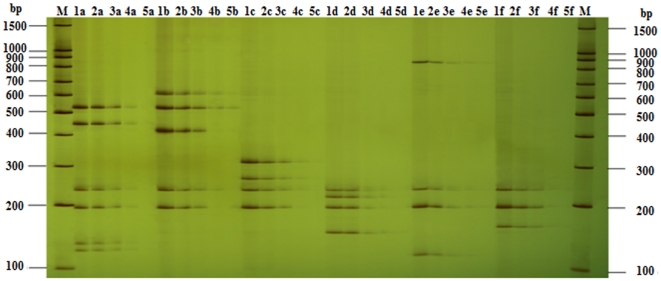
Sensitivity detection of single template by UP-M-PCR. a, MON 15985 of GM cotton; b, Huanong No. 1 of GM papaya; c, Bt11 of GM maize; d, GM rice with *bar* gene; e, GM canola with *hpt* gene; f, RRS of GM soybean. Lanes 1∼5, template concentration from 50, 5, 0.5, 0.1 ng to 0.05 ng; lane M, 100 bp DNA Marker.

The sensitivity of UP-M-PCR method was assayed in fifteen-plex PCR with only one DNA template. A set of UP-M-PCR reactions were performed with a series of change of template concentration from 50, 5, 0.5 and 0.1 ng to 0.05 ng, which resulted in significantly disproportionate amplification of target DNA by polyacrylamide gel electrophoresis assay. The amount of amplified products fell down along with the decrease of content of corresponding template, so did the intensity of bands. When the target template's amount was as low as 0.05 ng, there was no corresponding amplicon, so the detection limit of UP-M-PCR for target gene was 0.1 ng target DNA per reaction (Figure 5). Compared with the published conventional multiplex PCR system detecting eight GM maize lines at the same time [2], the limit of detection of which is 0.25% GM in the total 100 ng template, equaling to 0.25 ng GM content in the mixed samples, the novel PCR system has a much higher sensitivity.

### Application of UP-M-PCR in Detection as a Rapid Screening Method

**Figure 6:**
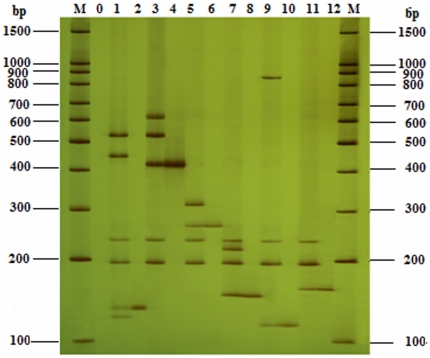
Application of UP-M-PCR. Lane 1,2, GM/non-GM cotton, with endogenous genes *sad1* (amplicon size 131 bp); Lane 3,4, GM/non-GM papaya, with endogenous genes *Pa* (amplicon size 403 bp); Lane 5,6, GM/non-GM maize, with endogenous genes *Ivr* (amplicon size 266 bp); Lane 7,8, GM/non-GM rice, with endogenous genes *sps* (amplicon size 150 bp); Lane 9,10, GM/non-GM canola, with endogenous genes *FatA* (amplicon size 116 bp); Lane 11,12, GM/non-GM soybean, with endogenous genes *Lec* (amplicon size 158 bp); Lane 0, negative control without template; lane M, 100 bp DNA Marker.

For most incidents, the detection samples are complicated by more than one GM material accompanied with multiple characteristics. Multiplexing provides a cost-effective diagnostic assay for GM detection with higher through-put and less consumption of samples and reagents as compared to simplex assays. The novel UP-M-PCR method was applied to test common GM and non-GM crops including cotton, papaya, maize, rice, canola and soybean, and there was significant difference between GMO and their non-GM parents (Figure 6).

## Discussion

The present paper has described the development and application of a novel UP-M-PCR method with an additional universal primer (UP) in the multiplex PCR reaction system. The designing of primers was very important on multiplex PCR techniques, because primer specificity and melting temperature (Tm) were more critical than conventional PCR. Comparing genomic DNA sequences of cotton, papaya, maize, rice, canola and soybean with the universal adapter sequence using DNAMAN, the universal adapter sequence were not matched with genomic DNA sequences. The process of selecting universal primer was showed in Table S1 and Figure S1. In optimized UP-M-PCR system, the universal primer has a concentration of 500 nmol L^−1^ at normal degree, while the concentration of every compound specific primer was as low as 1/50 to 1/10 of UP, therefore, the total amount of all the primers was almost equal to that of conventional singlet PCR and far less than that in conventional multiplex PCR, in which all the primers are mixed with a normal concentration about 500 nmol L^−1^. In a word, it really simplified the multiplex PCR reaction system, which was also the reason why it could circumvent the amplification disparity resulting from different primers in traditional multiplex PCR.

The choice of DNA polymerase is very important for the optimum performance of the PCR. The Phire™ Hot Start DNA polymerase, coupled with a preoptimized primer mix for different multiplex reactions, gave the best results both in terms of reproducibility and robustness. The use of hot start DNA polymerase prevents the formation of misprimed products and reduces primer-dimer formation. As the number of primers increases, the possible sequence dependent interactions between primers of different primer pairs also increase, which results in the formation of primer-dimers. Small differences in amplification efficiencies for the different primer pairs might result in the preferential amplification of some of the PCR products, leaving other PCR products at subdetectable levels. Hence, primer design, PCR cycling conditions, and the concentration of each reaction component need to be cautiously optimized in order to avoid the formation of primer-dimers and to detect all DNA targets simultaneously without any primer.

In most cases, agarose gel electrophoresis is used for the separation of DNA fragments ranging from 10 kb∼0.2 kb, but when separating particularly small pieces of DNA or small quantities of DNA, a vertical polyacrylamide gel is more appropriate, which has a higher resolution than that can be achieved with agarose gel electrophoresis. In this study, multiplex PCR products contained a series of DNA fragments in which most sizes were less than 500 bases and differed in size by only several bases (eg. *uidA* and *FatA*, only differed by 6 bases), and agarose gel electrophoresis cannot separated them efficiently. However, polyacrylamide gel has the advantage of exhibiting extraordinary resolution; pieces of DNA that differ in size by a single base pair can be separated in a polyacrylamide gel [20]. Thus sequencing gel electrophoresis (SGE) using polyacrylamide gel as separation medium, was chosen as the analysis tool for PCR amplicon fragments. What's more, the SGE system has longer lanes and higher separation voltage, which ensure the effective separation for DNA fragments of small difference. Primarily, SGE of DNA is only used for DNA sequencing. Nowadays its high resolution and large accommodation have made it widely used in many fields, such as RFLP, AFLP, SSR analysis and so on. The conditions of sequencing gel electrophoresis were carefully optimized in order to achieve best separating effect. The optimization of each factor was showed in Figure S3, S4, S5, S6. According to the optimal DNA resolution of different acrylamide concentration [21], the final acrylamide concentration is determined at 5%. The specified voltage is 1–8 volts/cm and centimeters in this case specifies the length of the gel from top to bottom (i.e the direction the DNA will travel). To avoid differential heating in the center of the gel which could cause poor image development, polyacrylamide gels were run at optimal constant power 40∼60 W (data not shown). The total electrophoresis time for complete separation of all amplicon fragments was about 90∼120 min depending on the running power.

To realize the potential of SGE, a visualization method offering superior clarity and sensitivity is also required. The silver staining method has proven very effective in this regard. As a method, silver staining was originally developed to detect proteins separated by polyacrylamide gels [22]. It was further optimized and applied to visualize other biological molecules e.g. nucleic acids [23], [24]. Silver staining of DNA has several advantages: (I) Image development and visualization is done under normal ambient light. Thus, the procedure can be performed entirely at the laboratory bench without the need for dark room or UV illumination facilities. (II) The image is resolved with the best possible sensitivity and detail, because silver is deposited directly on the molecules within the transparent gel matrix. Thus visualization is from the primary source and does not suffer any degradation or blurring that can accompany secondary imaging devices which involve fluorescence, autoradiography, focusing lenses, film development or digital image processing. (III) Silver staining offers similar sensitivity to autoradiography, but avoids radioactive handling, delays from development times and waste disposal issues [19]. Although this is an advantage in terms of scope, it nevertheless means that the protocol must be applied with due care; almost any other biological impurity such as stray human fingerprints incorporated into or onto the gel matrix on the gel surface will stain with perfect detail. It is thus important to use dust-free reagents of the best analytical grade, including the purest water available.

The UP-M-PCR method was originally developed by our lab and it firstly applied to the detection of stacked GM events Bt11×GA21 [19]. In this study we used the same PCR system and combined it with sequencing gel electrophoresis analysis to simultaneously detect 15 target genes in the GM crops successfully, but the sequence of UP is different from before, which just showed the flexibility of this novel PCR method. The key point of this approach is the idea of the reaction process, not a particular sequence of UP or some specific primers. Any sequence meeting the requirements of designing UP mentioned above can be applied in this PCR system. Otherwise, the careful optimization of the reaction system, including the choice of Tag Polymerase enzyme, the concentration of each primer, etc. is very significant for the high-throughout multiplex PCR detection system, which is also the important result of our study. What's more, the perfect combination of UP-M-PCR and sequencing gel electrophoresis, as well as the optimization of the electrophoresis conditions, is another important innovative point in this study.

With the dramatic expansion of global area under cultivation of GM crops, there is an urgent need to step up the development of robust, efficient, and reliable methods for GM detection. The developed UP-M-PCR method with sequencing gel electrophoresis analysis used to simultaneously detect nine commonly used selectable marker and reporter genes and six endogenous genes in a single reaction can be a reliable tool for the screening of GM crops and for unintentional mixing of GM seeds with non-GM seeds. Besides, the novel UP-M-PCR can be used in all the fields where multiplex PCR is needed, which has promising application future and is worth being popularized

## Materials and Methods

### Materials

The GM crops/events under study were all the main GM crops widely used nowadays, including GM maize event Bt11, GM cotton event MON 15985, GM rice with *bar* gene, GM papaya event Huanong No. 1, GM soybean event RRS and GM canola with *hpt* gene,along with their non-GM parent seeds of maize, cotton, rice, papaya, soybean and canola as controls. Before the extraction of DNA, they were ground respectively into powder with the size of 200 mesh in the fume hood in order to avoid cross-contamination.

### Preparation of DNA Template

Extract and purify genomic DNA from the finely ground powder samples described above, using the Wizard® Genomic DNA Purification Kit, according to the manufacturer's instructions. For specificity and sensitivity test, DNA was diluted in ddH_2_O to get a proper concentration. Evaluate the quality of the extracted DNA either on a 1% (wt/vol) agarose gel or by amplify a housekeeping gene by qualitative PCR, if the template DNA do not produce the expected amplicons, it suggests that the extracted DNA contains PCR inhibitors such as ethanol or xylene and these samples should not be used for further study.

### Designing of Primers

Comparing genomic DNA sequences of the common GM crops cotton, papaya, maize, rice, canola and soybean with the universal adapter sequence using DNAMAN, the universal adapter sequence were not matched with any genomic DNA sequences. For the amplification of *hpt*, *npt II*, *aadA*, *pat*, *bar*, *nos*, *Lec*, *sps*, *uidA* and *FatA* genes, published primer pairs [13], [25]–[27] were used. The universal primer (UP) and specific primer pairs for the amplification of *gus*, *Pa*, *Ivr*, *35s* and *sad1* genes were designed using ABI PRISM Primer Express Version 2.0 software(Applied Biosystems company, FosterCity, CA) with an optimal melting temperature(Tm) of about 60°C. When designing the UP primer, the factors including having binding sites with most GM crops genome as little as possible, being rich in GC contents and having a melting temperature (*Tm*) of about 60°C etc. were particularly considered to insure the suitability of the UP in the novel mutiplex PCR. Compound specific primers used in UP-M-PCR for the specific detection of these genes originate from the corresponding specific primers. Each compound specific primer contains a common sequence TTTGGTCGTGGTGGTGGTTT at its 5′-end, which is just the sequence of UP in this new PCR system. Details of primer sequences are shown in Table 1.

**Table 1:** Information of Compound Specific Primers Used in UP-M-PCR^a^.

Primer name	Primer sequence	Length (bp)	Ref.
hpt-839-F	*TTTGGTCGTGGTGGTGGTTT*CGCCGATGGTTTCTACAA	879	13
hpt-839-R	*TTTGGTCGTGGTGGTGGTTT*GGCGTCGGTTTCCACTAT		
gus-565-F	*TTTGGTCGTGGTGGTGGTTT*AAATCGCCGCTTTGGACATA	605	this study
gus-565-R	*TTTGGTCGTGGTGGTGGTTT*TTACTGGCTTTGGTCGTCATGA		
nptII-508-F	*TTTGGTCGTGGTGGTGGTTT*CCGACCTGTCCGGTGCCC	548	13
nptII-508-R	*TTTGGTCGTGGTGGTGGTTT*CCGCCACACCAGCCGGCC		
aadA-406-F	*TTTGGTCGTGGTGGTGGTTT*TCCGCGCTGTAGAAGTCACCATTG	446	13
aadA-406-R	*TTTGGTCGTGGTGGTGGTTT*CCGGCAGGCGCTCCATTG		
Pa-363-F	*TTTGGTCGTGGTGGTGGTTT*GGCTCAATATGGTATTCACTACAGAAAT	403	this study
Pa-363-R	*TTTGGTCGTGGTGGTGGTTT*CATCGGTTTTGGCTGCATAA		
pat-262-F	*TTTGGTCGTGGTGGTGGTTT*GAAGGCTAGGAACGCTTACG	302	13
pat-262-R	*TTTGGTCGTGGTGGTGGTTT*GCCAAAAACCAACATCATGC		
Ivr-226-F	*TTTGGTCGTGGTGGTGGTTT*TCCAAACTGAATCCGGTCTGA	266	this study
Ivr-226-R	*TTTGGTCGTGGTGGTGGTTT*GTGCGCTTCCTCTCGTTTTC		
35s-195-F	*TTTGGTCGTGGTGGTGGTTT*GCTCCTACAAATGCCATCATTGC	235	this study
35s-195-R	*TTTGGTCGTGGTGGTGGTTT*GATAGTGGGATTGTGCGTCATCCC		
bar-177-F	*TTTGGTCGTGGTGGTGGTTT*GCACAGGGCTTCAAGAGCGTGGTC	217	13
bar-177-R	*TTTGGTCGTGGTGGTGGTTT*GGGCGGTACCGGCAGGCTGAA		
nos-151-F	*TTTGGTCGTGGTGGTGGTTT*GTCTTGCGATGATTATCATATAATTTCTG	191	20
nos-151-R	*TTTGGTCGTGGTGGTGGTTT*CGCTATATTTTGTTTTCTATCGCGT		
Lec-118-F	*TTTGGTCGTGGTGGTGGTTT*GCCCTCTACTCCACCCCCA	158	21
Lec-118-R	*TTTGGTCGTGGTGGTGGTTT*GCCCATCTGCAAGCCTTTTT		
sps-110-F	*TTTGGTCGTGGTGGTGGTTT*GATCGCTTCCGCCATTAGCA	150	this study
sps-110-R	*TTTGGTCGTGGTGGTGGTTT*AACCGAGCGCGATCACTTGC		
sad1-91-F	*TTTGGTCGTGGTGGTGGTTT*CCACGAGACAGCCTATACCAAAA	131	22
sad1-91-R	*TTTGGTCGTGGTGGTGGTTT*CTTCTTCATCATGTCAGCAAATGC		
uidA-82-F	*TTTGGTCGTGGTGGTGGTTT*CACCACGGTGATATCGTCCAC	122	13
uidA-82-R	*TTTGGTCGTGGTGGTGGTTT* TTTCTTTAACTATGCCGGAATCCATC		
FatA-76-F	*TTTGGTCGTGGTGGTGGTTT*GGTCTCTCAGCAAGTGGGTGAT	116	this study
FatA-76-R	*TTTGGTCGTGGTGGTGGTTT*TCGTCCCGAACTTCATCTGTAA		
UP	*TTTGGTCGTGGTGGTGGTTT*		this study

a The table shows the details of primer sequences, expected DNA fragment length and the source of primer used in UP-M-PCR. Each primer pair originates from the corresponding specific primer set (sequence in straight matter) and has a common sequence TTTGGTCGTGGTGGTGGTTT (20 bp) at its 5′ - end in italics, which is also the sequence of the universal primer (UP) used in this developed new way.

### Universal Primer-Multiplex PCR (UP-M-PCR)

**Figure 7:**
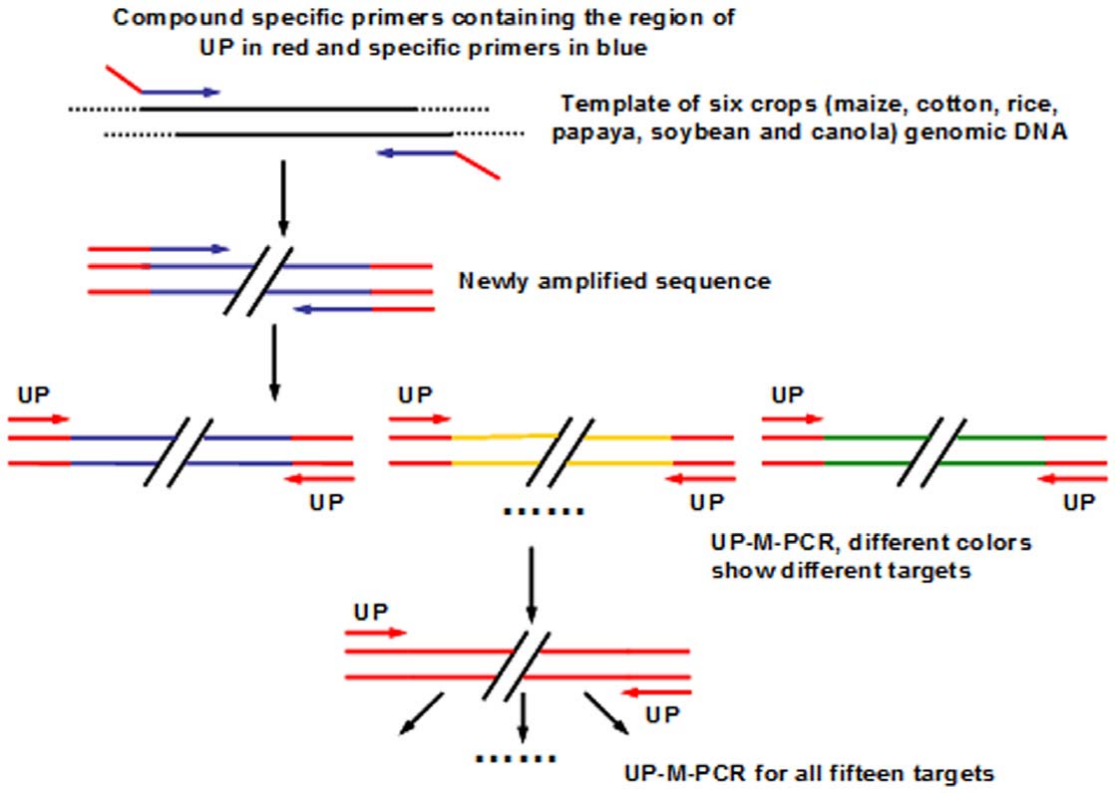
Amplification routine of UP-M-PCR. Each compound specific primer contained a universal sequence at the 5′-end (red) and the specific primer at the 3′-end (blue). The amplified fragments with the primer pairs of different targets are individually marked in different colors. The amplified fragments only by the universal primer are marked in red.

Compared with traditional multiplex PCR system, UP-M-PCR contains more of a universal primer (UP) at a normal concentration (500 nmol/L), a little quantity (10∼50 nmol/L) of 15 compound specific primers targeting for the 15 marker and endogenous genes. All the primers include a common sequence at its 5′-end, which is also the sequence of the UP. The amplification routine of UP-M-PCR is shown in Figure 7. At the initial stage of the reaction (about the former ten cycles), the compound specific primers take main action for amplification of target sequences due to their higher annealing temperature, while the universal primer almost has no amplification. With the compound specific primers used up and the amplified products incorporating the UP adaptor increasing, the UP begins to play a leading role to take the amplicons as templates and shows its ability to amplify the fragments of seven different targets.

### PCR Conditions

PCR analyses were carried out on Peltier Thermal Cycler Controller (MJ Research, BioRad Laboratories, Mass., U.S.A.). The efficiency of the primer pairs to amplify the target sequences was tested by simplex PCR using the corresponding genomic DNA with different target genes.

The concentrations of each primer were optimized in the single and multiplex PCR system, ranging from 500 to 5 nmol L^−1^ with series of dilutions, while the universal primer always had a normal concentration of 500 nmol L^−1^. The final optimized compound specific primers mix contains 10 nM of 35s-195-F/R and nos-151-F/R (about 1/50 of UP), 16 nM of nptII-508-F/R and Pa-363-F/R (about 1/30 of UP), 50 nM of hpt-839-F/R, Ivr-262-F/R and Lec-110-F/R (about 1/10 of UP), and 25 nM of all other primers, including gus-565-F/R, aadA-406-F/R, pat-262-F/R, bar-177-F/R, sps-110-F/R, sad1-91-F/R, uidA-82-F/R and FatA-76-F/R (about 1/20 of UP).

To test the efficiency of Taq Polymerase to be employed in PCR assays, comparative tests were made with several Taq polymerases, such as Phire™ Hot Start DNA polymerase, iProof™ High-Fidelity DNA polymerase, and *TaKaRa Taq*™. All multiplex PCR assays were performed in a final volume of 80 µL with the following reagent concentrations: 250 ng template DNA mix, 5×Hot Start PCR buffer, 0.2 mM of dNTPs, 500 nmol/L universal primer, 1 µL DNA polymerase and the preoptimized compound specific primers mix.

To choose the best annealing temperature, a gradient PCR with annealing temperatures ranging from 56 to 64°C was performed. The extension temperature and the time for annealing and extension were also optimized in order to choose the best PCR conditions. The final amplification conditions were initial denaturation at 95°C for 10 min, 40 cycles consisting of denaturation at 95°C for 30 s, primer annealing at 60°C for 50 s, primer extension at 70°C for 50 s, and final extension at 70°C for 10 min. Each reaction was run in triplicate.

### Analysis of PCR Products

PCR products were analyzed on sequencing gel electrophoresis (SGE). The gel was prepared with 5% polyacrylamide (acrylamide/bisacrylamide, 29/1; containing 3.5 M urea). Polyacrylamide gels are poured and run in 1× TBE at constant power 40∼60 W to avoid differential heating in the center of the gel. After electrophoresis, silver staining method used to visualize DNA fragments, and images were recorded with a digital camera.

## Supporting Information

Figure S1 **Selection of universal primers.** A, B, C: Reaction system adding UP1, UP2 and UP3 respectively. Lane 1a/1b, 2a/2b, 3a/3b: duplex PCR for amplifying hpt/pat, hpt/nptII, nptII/pat; lane 4a/4b: triplex PCR for amplifying hpt/nptII/pat; lane 1c/2c/3c/4c: NTC (no template control); lane M: DNA Marker DL 2000.
(DOC)

Figure S2 **Optimization of primer concentration for UP-M-PCR.** (A) UP-M-PCR for amplifying *hpt*, *gus*, *nptII*, *aadA*, *Pa*, *pat* and *Ivr* gene. Lane 1–4: after concentration adjustment; lane 5–8: before concentration adjustment. (B) UP-M-PCR for amplifying *35s*, *bar*, *nos* and *Lec* gene. Lane 1–3: after concentration adjustment; lane 4–6: before concentration adjustment. (C) UP-M-PCR for amplifying *sps*, *uidA*, *sad1* and *FatA* gene. Lane 1–3: after concentration adjustment; lane 4–6: before concentration adjustment; lane M: DNA Marker DL 2000.
(DOC)

Figure S3 **Determination of electrophoretic condition.** A: Constant voltage of 500 V; B: Constant Power of 60 W.
(DOC)

Figure S4 **Determination of gel's concentration.** A, B, C: Gel's concentration of 4%, 5%, 6%, respectively.
(DOC)

Figure S5 **Determination of the urea concentration.** A, B, C: Gel's concentration of 5%, with the urea concentration of 0, 7 mol/L, 3.5 mol/L, respectively.
(DOC)

Figure S6 **Determination of sample treatment.** A: Sample treated by denaturalization. B: Sample not treated by denaturalization.
(DOC)

Table S1 **Primers for selecting universal primer^a^.**
(DOC)
