# Supplementary material for: A Novel Universal Primer-Multiplex-PCR Method with Sequencing Gel Electrophoresis Analysis
Source: PLoS One. 2012 Jan 17;7(1):e22900. doi: 10.1371/journal.pone.0022900 (PMC3260127; doi:10.1371/journal.pone.0022900)
Supplement: Figure S1 — Selection of universal primers. A, B, C: Reaction system adding UP1, UP2 and UP3 respectively. Lane 1a/1b, 2a/2b, 3a/3b: duplex PCR for amplifying hpt/pat, hpt/nptII, nptII/pat; lane 4a/4b: triplex PCR for amplifying hpt/nptII/pat; lane 1c/2c/3c/4c: NTC (no template control); lane M: DNA Marker DL 2000. (DOC) [file pone.0022900.s001.doc]

**Selecting universal primer**

The universal primer was designed using the ABI Prism Primer Express Software by our own, and when designing the factors including having the binding sites with most GM crops genome as little as possible, being rich in GC contents and having a melting temperature (*Tm*) of about 60 °C etc. According to these principles, three universal primers were designed. In order to select the optimum sequence for the study, three gene-specific primers, including *hpt*, *nptII* and *pat*, with three universal sequences at their 5’ ends respectively were used in UP-M-PCR systems with three universal primers in them. The information of primers and the amplification results were showed in Figure S1.


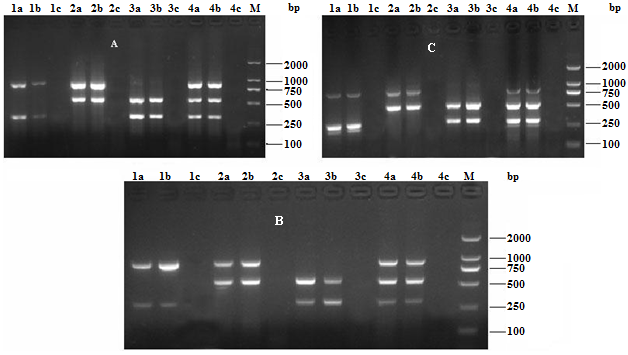


Figure S1 Selection of universal primers

A、B、C: Reaction system adding UP1, UP2 and UP3 respectively. Lane 1a/1b, 2a/2b, 3a/3b: duplex PCR for amplifying *hpt* / *pat*, *hpt* / *nptII*, *nptII* / *pat*; lane 4a/4b: triplex PCR for amplifying *hpt* / *nptII* / *pat*; lane 1c/2c/3c/4c: NTC (no template control); lane M: DNA Marker DL 2000.

It can be seen that UP-M-PCR system with UP2 (Figure S1, B) had the best amplicons and there were not any non-specific products, so UP2 with the sequence 5’-TTTGGTCGTGGTGGTGGTTT-3’ were chosen as the universal primer for the 15-plex PCR reaction.
