# Supplementary material for: A Novel Universal Primer-Multiplex-PCR Method with Sequencing Gel Electrophoresis Analysis
Source: PLoS One. 2012 Jan 17;7(1):e22900. doi: 10.1371/journal.pone.0022900 (PMC3260127; doi:10.1371/journal.pone.0022900)
Supplement: Figure S2 — Optimization of primer concentration for UP-M-PCR. (A) UP-M-PCR for amplifying hpt, gus, nptII, aadA, Pa, pat and Ivr gene. Lane 1–4: after concentration adjustment; lane 5–8: before concentration adjustment. (B) UP-M-PCR for amplifying 35s, bar, nos and Lec gene. Lane 1–3: after concentration adjustment; lane 4–6: before concentration adjustment. (C) UP-M-PCR for amplifying sps, uidA, sad1 and FatA gene. Lane 1–3: after concentration adjustment; lane 4–6: before concentration adjustment; lane M: DNA Marker DL 2000. (DOC) [file pone.0022900.s002.doc]

**Optimization of primer concentration**

In order to optimize the concentration of each compound specific primers, all 15 primer pairs were divided into three groups according to the amplicon size: ① *hpt , gus , nptII , aadA , Pa , pat , Ivr* (1000~250bp)；② *35s , bar , nos, Lec* (250~150bp)；③ *sps , uidA, sad1 , FatA* (150~100bp). In each group, primers were first mixed at 25 nmol L-1, and then there was an adjustment according to the amplification results, while the universal primer maintained at 500 nmol L-1, which ensure that each primer had its amplified products. The results were showed in Figure S2.


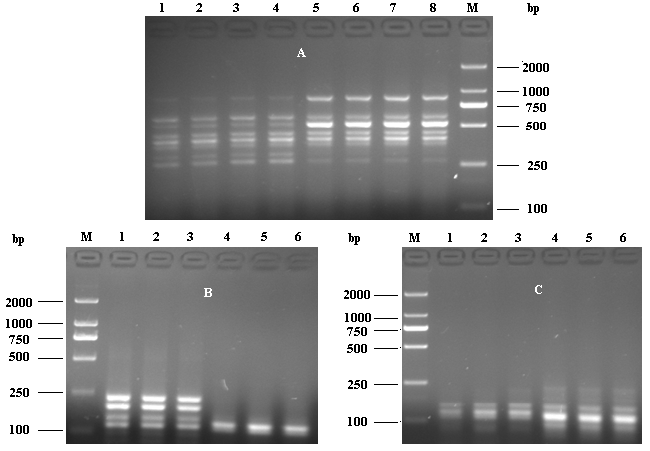


Figure S2 Optimization of primer concentration for UP-M-PCR

(A) UP-M-PCR for amplifying *hpt*, *gus*, *nptII*, *aadA*, *Pa*, *pat* and *Ivr* gene. Lane 1-4: after concentration adjustment; lane 5-8: before concentration adjustment. (B) UP-M-PCR for amplifying *35s*, *bar*, *nos* and *Lec* gene. Lane 1-3: after concentration adjustment; lane 4-6: before concentration adjustment. (C) UP-M-PCR for amplifying *sps*, *uidA*, *sad1* and *FatA* gene. Lane 1-3: after concentration adjustment; lane 4-6: before concentration adjustment; lane M: DNA Marker DL 2000.
