# Supplementary material for: A Novel Universal Primer-Multiplex-PCR Method with Sequencing Gel Electrophoresis Analysis
Source: PLoS One. 2012 Jan 17;7(1):e22900. doi: 10.1371/journal.pone.0022900 (PMC3260127; doi:10.1371/journal.pone.0022900)
Supplement: Figure S3 — Determination of electrophoretic condition. A: Constant voltage of 500 V; B: Constant Power of 60 W. (DOC) [file pone.0022900.s003.doc]

**Determination of** **conditions for sequencing gel electrophoresis**

The conditions of sequencing gel electrophoresis were carefully optimized in order to achieve best separating effect. ① Electrophoretic condition. We used constant voltage and constant power respectively to run electrophoresis. The banding pattern of constant power was much better. ② Gel’s concentration. As the optimal separation range of 5% polyacrylamide gel is 80~500bp (Joseph S, David W R. Molecular Cloning: A Laboratory Manual. 3rd ed. Beijing: Science Press, 2002), which is close to the size of amplicons, so we compared the effect of 4%, 5% and 6% polyacrylamide gel. Results showed that 5% polyacrylamide gel is still the best choice for separation. ③ Concentration of urea in gel. Non-denatured gel without urea, denatured gel with 7 mol L^-1^ urea and denatured gel with 3.5 mol L^-1^ urea were used respectively, and denatured gel with 3.5 mol L^-1^ urea was chosen for the best results. ④ Sample treatment. Samples treated by denaturalization or not were loaded respectively, and the results showed that samples without denaturalization appeared much clearer and correct band. All the results above showed in Figure S3-S6.


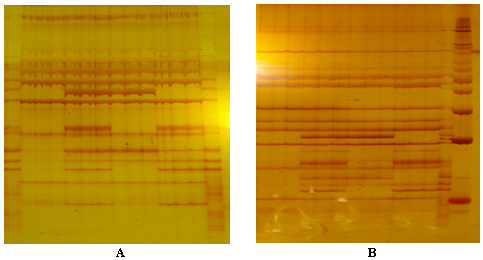


Figure S3 Determination of electrophoretic condition

A: Constant voltage of 500V; B: Constant Power of 60W.
