# Supplementary figures and images for: A Novel Universal Primer-Multiplex-PCR Method with Sequencing Gel Electrophoresis Analysis
Source: PLoS One. 2012 Jan 17;7(1):e22900. doi: 10.1371/journal.pone.0022900 (PMC3260127; doi:10.1371/journal.pone.0022900)

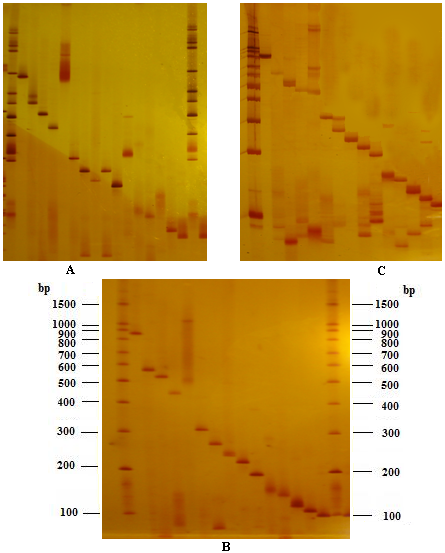


Figure S4 Determination of gel’s concentration

A、B、C：Gel’s concentration of 4%、5%、6%, respectively.

Supplement: Figure S4 — Determination of gel's concentration. A, B, C: Gel's concentration of 4%, 5%, 6%, respectively. (DOC) [file pone.0022900.s004.doc]
