# Supplementary material for: A Novel Universal Primer-Multiplex-PCR Method with Sequencing Gel Electrophoresis Analysis
Source: PLoS One. 2012 Jan 17;7(1):e22900. doi: 10.1371/journal.pone.0022900 (PMC3260127; doi:10.1371/journal.pone.0022900)
Supplement: Figure S5 — Determination of the urea concentration. A, B, C: Gel's concentration of 5%, with the urea concentration of 0, 7 mol/L, 3.5 mol/L, respectively. (DOC) [file pone.0022900.s005.doc]

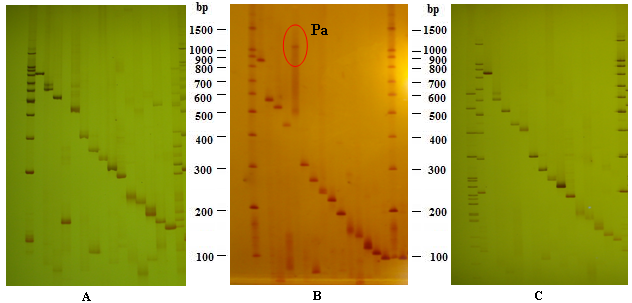


Figure S5 Determination of the urea concentration

A、B、C：Gel’s concentration of 5%, with the urea concentration of 0, 7 mol/L, 3.5 mol/L, respectively.

.
