# Supplementary material for: A Novel Universal Primer-Multiplex-PCR Method with Sequencing Gel Electrophoresis Analysis
Source: PLoS One. 2012 Jan 17;7(1):e22900. doi: 10.1371/journal.pone.0022900 (PMC3260127; doi:10.1371/journal.pone.0022900)
Supplement: Figure S6 — Determination of sample treatment. A: Sample treated by denaturalization. B: Sample not treated by denaturalization. (DOC) [file pone.0022900.s006.doc]

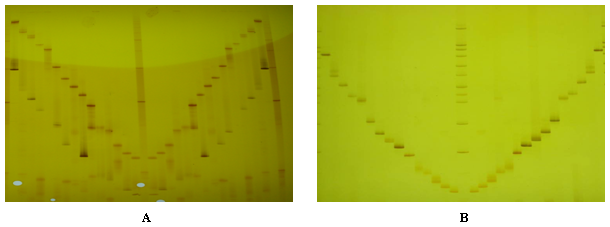


Figure S6 Determination of sample treatment

A: Sample treated by denaturalization. B: Sample not treated by denaturalization.
