# Supplementary material for: A Novel Universal Primer-Multiplex-PCR Method with Sequencing Gel Electrophoresis Analysis
Source: PLoS One. 2012 Jan 17;7(1):e22900. doi: 10.1371/journal.pone.0022900 (PMC3260127; doi:10.1371/journal.pone.0022900)
Supplement: Table S1 — Primers for selecting universal primera. (DOC) [file pone.0022900.s007.doc]

Table S1 Primers for selecting universal primer**^a^**

| Target gene | Primer name | Sequence（5′ →3′ ） | Length  (bp) |
| --- | --- | --- | --- |
| *hpt* | UP1-hpt-F | *AACCAGACCAGACCACGGACC* CGCCGATGGTTTCTACAA | 881 |
|  | UP1-hpt-R | *AACCAGACCAGACCACGGACC* GGCGTCGGTTTCCACTAT |  |
|  | UP2-hpt-F | *TTTGGTCGTGGTGGTGGTTT* CGCCGATGGTTTCTACAA | 879 |
|  | UP2-hpt-R | *TTTGGTCGTGGTGGTGGTTT* GGCGTCGGTTTCCACTAT |  |
|  | UP3-hpt-F | *CCTACGACTCGAGCAACCCC* CGCCGATGGTTTCTACAA | 879 |
|  | UP3-hpt-R | *CCTACGACTCGAGCAACCCC* GGCGTCGGTTTCCACTAT |  |
| *nptII* | UP1-nptII-F | *AACCAGACCAGACCACGGACC* CCGACCTGTCCGGTGCCC | 550 |
|  | UP1-nptII-R | *AACCAGACCAGACCACGGACC* CCGCCACACCAGCCGGCC |  |
|  | UP2-nptII-F | *TTTGGTCGTGGTGGTGGTTT* CCGACCTGTCCGGTGCCC | 548 |
|  | UP2-nptII-R | *TTTGGTCGTGGTGGTGGTTT* CCGCCACACCAGCCGGCC |  |
|  | UP3-nptII-F | *CCTACGACTCGAGCAACCCC* CCGACCTGTCCGGTGCCC | 548 |
|  | UP3-nptII-R | *CCTACGACTCGAGCAACCCC* CCGCCACACCAGCCGGCC |  |
| *pat* | UP1-pat-F | *AACCAGACCAGACCACGGA* CCGAAGGCTAGGAACGCTTACG | 304 |
|  | UP1-pat-R | *AACCAGACCAGACCACGGA* CCGCCAAAAACCAACATCATGC |  |
|  | UP2-pat-F | *TTTGGTCGTGGTGGTGGTTT* GAAGGCTAGGAACGCTTACG | 302 |
|  | UP2-pat-R | *TTTGGTCGTGGTGGTGGTTT* GCCAAAAACCAACATCATGC |  |
|  | UP3-pat-F | *CCTACGACTCGAGCAACCCC* GAAGGCTAGGAACGCTTACG | 302 |
|  | UP3-pat-R | *CCTACGACTCGAGCAACCCC* GCCAAAAACCAACATCATGC |  |

**^a^**: Sequence in italics are universal sequence.
